# Erythrocyte Membrane Nanomechanical Rigidity Is Decreased in Obese Patients †

**DOI:** 10.3390/ijms23031920

**Published:** 2022-02-08

**Authors:** Jesús Sot, Aritz B. García-Arribas, Beatriz Abad, Sara Arranz, Kevin Portune, Fernando Andrade, Alicia Martín-Nieto, Olaia Velasco, Eunate Arana, Itziar Tueros, Carla Ferreri, Sonia Gaztambide, Félix M. Goñi, Luis Castaño, Alicia Alonso

**Affiliations:** 1 Instituto BIOFISIKA (CSIC, UPV/EHU), Departamento de Bioquímica, Universidad del País Vasco, 48940 Leioa, Spain; jesussot@hotmail.com (J.S.); aritzgarciaar@hotmail.com (A.B.G.-A.); felix.goni@ehu.es (F.M.G.); 2 SGIKER, Servicios Generales de Investigación (SGiker), Universidad del País Vasco, 48940 Leioa, Spain; beatriz.abad@ehu.es; 3 AZTI, Food Research, Basque Research and Technology Alliance (BRTA), Parque Tecnológico de Bizkaia, Astondo Bidea, Edificio 609, 48160 Derio, Spain; sarranz@azti.es (S.A.); kportune@gmail.com (K.P.); itueros@azti.es (I.T.); 4 Biocruces Bizkaia, Hospital Universitario Cruces, CIBERDEM, CIBERER, Endo-ERN, UPV-EHU, 48903 Barakaldo, Spain; fernando.andrade@osakidetza.eus (F.A.); alicia.martinnieto@osakidetza.eus (A.M.-N.); olaia.velasco@osakidetza.eus (O.V.); eunate.arana@osakidetza.eus (E.A.); mariasonia.gaztambidesaenz@osakidetza.eus (S.G.); luisantonio.castanogonzalez@osakidetza.eus (L.C.); 5 ISOF, Consiglio Nazionale delle Ricerche, Via Piero Gobetti, 101, 40129 Bologna, Italy; carla.ferreri@isof.cnr.it

**Keywords:** obesity, cell membrane physical properties, membrane fluidity, fluorescence polarization, atomic force microscopy, membrane breakthrough force, lipidomics

## Abstract

This work intends to describe the physical properties of red blood cell (RBC) membranes in obese adults. The hypothesis driving this research is that obesity, in addition to increasing the amount of body fat, will also modify the lipid composition of membranes in cells other than adipocytes. Forty-nine control volunteers (16 male, 33 female, BMI 21.8 ± 5.6 and 21.5 ± 4.2 kg/m^2^, respectively) and 52 obese subjects (16 male and 36 female, BMI 38.2± 11.0 and 40.7 ± 8.7 kg/m^2^, respectively) were examined. The two physical techniques applied were atomic force microscopy (AFM) in the force spectroscopy mode, which allows the micromechanical measurement of penetration forces, and fluorescence anisotropy of trimethylammonium diphenylhexatriene (TMA-DPH), which provides information on lipid order at the membrane polar–nonpolar interface. These techniques, in combination with lipidomic studies, revealed a decreased rigidity in the interfacial region of the RBC membranes of obese as compared to control patients, related to parallel changes in lipid composition. Lipidomic data show an increase in the cholesterol/phospholipid mole ratio and a decrease in sphingomyelin contents in obese membranes. ω-3 fatty acids (e.g., docosahexaenoic acid) appear to be less prevalent in obese patient RBCs, and this is the case for both the global fatty acid distribution and for the individual major lipids in the membrane phosphatidylcholine (PC), phosphatidylethanolamine (PE) and phosphatidylserine (PS). Moreover, some ω-6 fatty acids (e.g., arachidonic acid) are increased in obese patient RBCs. The switch from ω-3 to ω-6 lipids in obese subjects could be a major factor explaining the higher interfacial fluidity in obese patient RBC membranes.

## 1. Introduction

Obesity is defined by the World Health Organization as abnormal or excessive fat accumulation that presents a risk to health. More than 2.1 billion people—nearly 30 percent of the global population—are overweight or obese according to updated data from NCD-RisC 2017 [1]. Initiatives to curb this pandemic include actions toward maternal, infant and young child nutrition, as well as strategies on diet and physical exercise. A rational approach to improve this major form of disease should include, together with the epidemiological and educational activities, an improved understanding of the underlying molecular mechanisms. According to the Endocrine Society, there is “growing evidence suggesting that obesity is a disorder of the energy homeostasis system, rather than simply arising from the passive accumulation of excess weight” [2]. Lipids, or fats, are the molecules with the highest energetic content in the biosphere, thus it is understandable that they have been the target of many dietary interventions and of as many metabolic studies on obesity in the past [3].

Among lipids, triglycerides are by far the most abundant class of lipids in the human body, making up the bulk of the so-called “body fat” [4]. They are also the main lipids in food, including vegetable oils [5]. Membrane lipids, mainly phospholipids and cholesterol, constitute a small fraction of the human body lipids. Cholesterol and its oxidation products are well-known pathogenic agents in cardiovascular disease, but they also serve as essential membrane lipids and metabolites [6,7]. The most abundant plasma membrane lipids are phospholipids, organized in a double layer, or bilayer, that constitutes the membrane matrix [8]. Based on the observation that the composition of fatty acid residues in phospholipids is typical for each tissue [9], attention to the membrane lipidome has increased in health and disease [10], also in connection with the “membrane lipid therapy” [11].

Research in obesity has concerned mostly blood plasma, focusing on the circulating lipids and lipoproteins [12,13,14]. Compared with the massive amount of investigations on triglycerides and obesity, relatively few studies have dealt with membrane phospholipids in body weight alterations. The main hypothesis driving our research is that obesity, in addition to increasing the amount of body fat, will also modify the lipid composition of membranes in cells other than adipocytes. A few studies have dealt with this subject in the past. Pan et al. [15] observed an increased Δ9-desaturase (thus enhanced fatty acid unsaturation) and a lowered Δ5-desaturase (thus decreased synthesis of polyunsaturated fatty acids, or PUFA) in obese patients. Min et al. [16], in red blood cells (RBC) from patients with gestational diabetes, often linked to obesity, described a decrease in the phospholipid phosphatidylethanolamine (PE) and arachidonic (20:4, AA) and docosahexaenoic (22:6, DHA) acids. Cazzola et al. [17] reported on an increase in the cholesterol/phospholipid ratio in RBC membranes from obese patients, together with a decrease in ω-3 fatty acids (e.g., DHA) and an increase in ω-6 (e.g., AA). In a detailed study of twin pairs discordant for obesity, Pietiläinen et al. [18] found increased proportions of palmitoleic acid (16:1) and AA, together with increased levels of ethanolamine plasmalogens in adipose tissue of the obese twins. More recently, studies from this consortium have found that, in obese child RBC membranes, AA as well as saturated and *trans*-unsaturated fatty acyl chains were increased [19]. In a parallel study, AA was increased and monounsaturated chains were decreased in obese children [20].

Fatty acids can change membrane biophysical properties [21], thus influencing membrane-associated processes like protein–lipid interactions, enzymatic activity, and regulation of surface receptors [22,23]; therefore, it is significant to foster an interdisciplinary approach to understand the relationship between disease-induced membrane lipidome composition and properties. The present contribution deals with the physical properties of RBC membranes in normal and obese adults. The two main techniques applied are atomic force microscopy (AFM) in the force spectroscopy mode, which allows the micromechanical measurement of penetration forces [24,25], and fluorescence anisotropy, which provides information on membrane lipid order [26]. The probe used in fluorescence studies has been trimethylammonium diphenylhexatriene (TMA-DPH) that, in contrast to the more commonly used DPH, becomes located and provides information on the interfacial bilayer region between the phospholipid hydrophobic chains and polar head groups [27]. The combination of nanomechanical, spectroscopic, and lipidomic studies reveals a decreased rigidity in the interfacial region of the RBC membranes of obese as compared to control patients, related to parallel changes in lipid composition.

## 2. Results

### 2.1. Patient Recruitment and Anthropometry

A total of 101 volunteers were recruited through a public call. Details can be found in Table 1. Forty-nine control volunteers (16 male and 33 female, mean age 30.2 and 34.5 years, BMI 21.8 ± 5.6 and 21.5 ± 4.2 kg/m^2^, respectively) and 51 obese subjects (16 male and 36 female, mean age 48 and 45.8 years, BMI 38.2 ± 11.0 and 40.7 ± 8.7 kg/m^2^, respectively) were examined. The study protocol was approved by the Basque Country Clinical Research Ethics Committee (permission number PI2019219) and carried out according to the Declaration of Helsinki Good Clinical Practice guidelines. Subjects under study were included after acceptance to participate in the study and signing of informed consent.

### 2.2. Obese Patient RBC Membranes Exhibit Lower Nanomechanical Rigidity under the Atomic Force Microscope

**Figure 1:**
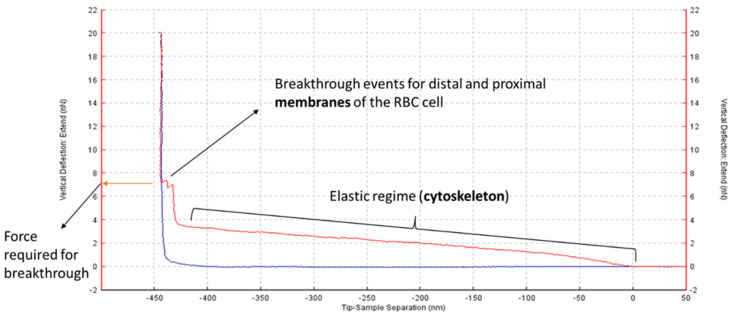
Representative AFM force–distance curve of an RBC. The AFM tip performs an indentation process on a supported RBC, initiated from X = 0 along the red line (trace), up to the maximum force (20 nN in this case), and coming back to the initial position long the blue line (retrace). The trace line has three distinct phases: (i) first, an elastic deformation of the cell occurs (the force required for this process depends on the cell cytoskeleton); (ii) then, after further compression, the AFM tip pierces immediately through both RBC membranes (distal and proximal); and (iii) finally the tip achieves maximum force against the support, without further X-axis displacement. Membrane rupture is achieved at a definite force, marked by the sudden appearance of small peaks, at a Y-axis value that can be statistically quantitated (performing 50–75 curves for each sample). These experiments were performed at room temperature.

Atomic Force Microscopy (AFM) is a powerful tool that allows, in the force spectroscopy mode, direct measurements of mechanical resistance of any surface when the tip approaches it with a definite force, a process called ‘indentation’. In this case, given the small thickness of RBCs (~1 µm), our approach was to fully pierce through the cells by performing a maximum force of approximately 10–20 nN. Thus, we were able to measure and quantify membrane breakthrough events for the RBC membrane, with each curve typically showing two different sequential rupture events as the cell was completely pierced (an example can be seen in Figure 1), as mentioned in the Materials and Methods section.

**Figure 2:**
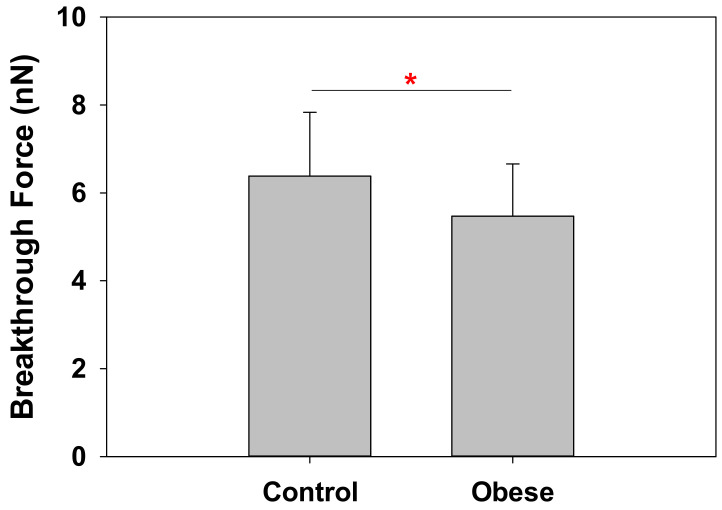
AFM force spectroscopy experiments on RBC. These measurements were performed at room temperature. Obese patient RBC are significantly less resistant to AFM punch-through experiments, pointing to a decrease in stiffness (number of patients *n* = 20 for control, *n* = 22 for obese; 50–75 measurements for each patient). Average values ± S.D. (*) Significance according to Student’s t-test: *p* = 0.03.

Results shown in Figure 2 for both control and obese patient RBC indicate a significant decrease in RBC stiffness for obese patients. Nanomechanical resistance values were 6.38 ± 1.45 nN for control RBC and 5.47 ± 1.19 nN for obese patient RBC (*p* = 0.03).

### 2.3. Anisotropy at the Polar–Nonpolar Interface Is Decreased in Obese Patient Erythrocyte Membranes

Anisotropy is defined as the capacity of a material (in this case, a molecule) to exhibit different properties in different directions. Anisotropic optical probes can be used, therefore, to test the fluidity of an environment: if we consider a lipophilic anisotropic probe, its mobility will be directly related to the degree of molecular order of the membrane in the area in which the probe is embedded. In general, anisotropy values are decreased when the membrane is more fluid. With an increased fluidity, both molecular order and microviscosity decrease, causing higher rotational diffusion of the probe [28]. In our case, a TMA-DPH probe was used to locally evaluate the fluidity in a particular zone of RBC membranes, namely, the space near the hydrophilic–hydrophobic interface (i.e., the boundary between polar headgroups and lipid tails). TMA-DPH anisotropy was measured at two temperatures, 20 °C and 37 °C, the latter chosen due to its physiological relevance.

**Figure 3:**
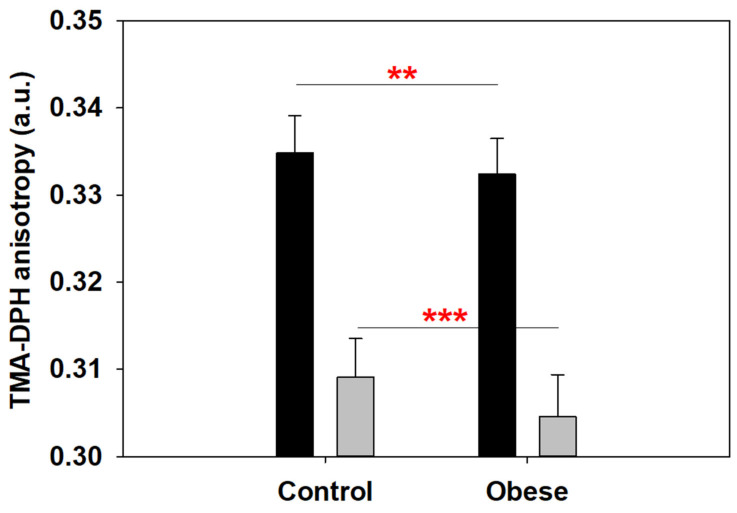
TMA-DPH anisotropy measurements of RBC membranes. The black bars represent measurements at 20 °C, while gray ones represent those at 37 °C. At both temperatures, a clear decrease for anisotropy values was detected for obese patient RBC, which indicates a higher membrane fluidity (*n* = 49 for control, *n* = 52 for obese). Average values ± S.D. Significance according to Student’s t-test: (**) *p* < 0.01; (***) *p* < 0.001.

TMA-DPH probe anisotropy measurements revealed a significant reduction in membrane order for obese patient RBCs when compared to control RBCs (Figure 3). Interestingly, this occurred for both 20 °C and 37 °C data sets, and the difference was even more significant at 37 °C, with a global decrease of anisotropy values as membranes become more fluid due to the increase in T. These results point to a localization of membrane fluidity difference in obese patient RBC close to the space between the polar headgroups and the lipid tails. The above measurements, taken at room temperature (~20 °C), show a good correlation with AFM increased penetrability (decreased rigidity) results, and point to a reduction in overall RBC membrane stiffness in obese patients. This will be further elaborated in the Discussion section.

### 2.4. Lipidomics Reveal an Altered Metabolism of Sphingomyelin (SM), ω-6 and ω-3 Fatty Acids

**Figure 4:**
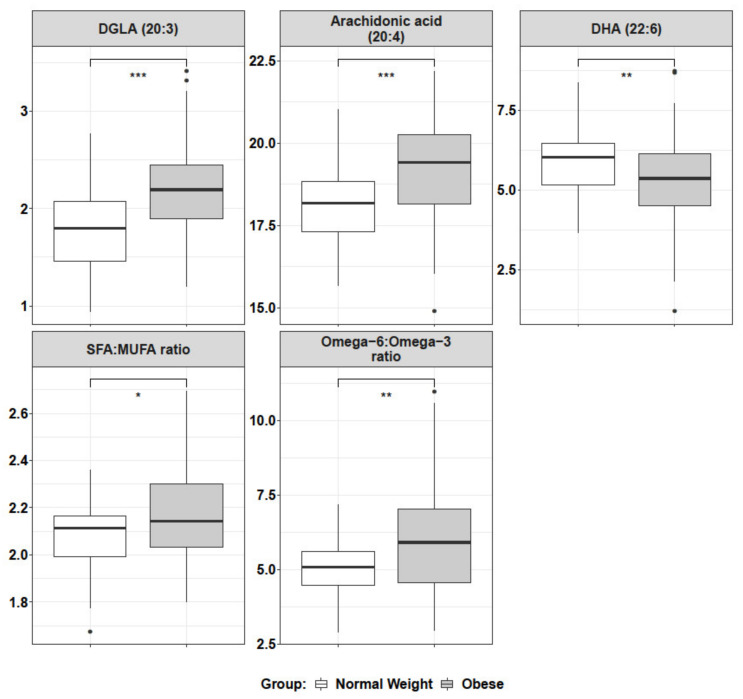
Lipidomic quantitation of global fatty acid presence in mature RBCs. Empty boxes refer to control (normal weight) group, while gray boxes represent obese patients. Significant differences are detected for dihomo-γ-linolenic acid (DGLA), arachidonic acid, DHA levels, SFA/MUFA, and ω-6/ω-3 ratios, pointing to a metabolic switch for obese patient RBC membranes. Significance according to Student’s t-test: (*) *p* < 0.05; (**) *p* < 0.01; (***) *p* < 0.001. (*n* = 49 for control, *n* = 52 for obese).

In order to unveil the possible metabolic causes of the observed decreased RBC rigidity in obese patients, a lipidomic analysis of their RBC was performed. These experiments included the study of global fatty acids as well as that of specific lipid classes. Global fatty acid lipidomics of mature RBCs (Figure 4) showed a small but significant increase of the saturated/monounsaturated fatty acid (SFA/MUFA) ratio for obese patient RBCs, which implies that obese RBCs have either less saturated fatty acids or more monounsaturated ones. In addition, the polyunsaturated fatty acid (PUFA) profile of omega-6 (ω-6) and omega-3 (ω-3) fatty acids indicated that the ω-6/ω-3 ratio was increased for obese patient RBCs, pointing to a combined increase of ω-6 and a decrease of ω-3 for obese patient RBCs. A further analysis of some common PUFA such as arachidonic acid (ω-6) and its precursor DGLA (dihomo-γ-linolenic acid) (ω-6) and DHA (docosahexaenoic acid) (ω-3) pointed in the same direction, as obese patient RBCs exhibited a significant increase in arachidonic acid and DGLA, while DHA was decreased.

**Figure 5:**
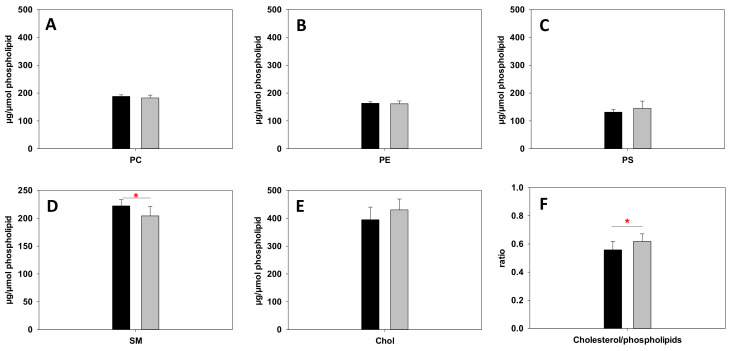
Lipidomic quantitation of specific lipid species in RBC. Species studied were PC (**A**), PE (**B**), PS (**C**), SM (**D**), and Chol (**E**). Chol/total phospholipid mol ratio is shown in panel (**F**). Black bars refer to control RBC group, while gray bars represent the obese patient RBC group. A significant decrease in SM and an increase in Chol/phospholipid ratio were detected for obese patient RBC. Average values ± S.D. Significance according to Student’s t-test: (*) *p* < 0.05. *n* = 8.

Lipid classes were then examined by separation using UHPLC coupled with mass characterization by tandem MS, as described in the Experimental Section. Lipidomics results for the percent distribution of individual lipid classes are shown in Figure 5, namely, phospholipids (PC, PE, PS, and sphingomyelin SM, the latter also a sphingolipid) and the most abundant lipid in the RBC membrane, cholesterol (Chol), as well as the Chol/total phospholipid mol ratio. A significant decrease in SM was detected in obese patient RBCs (Figure 5D), while the other lipid species did not exhibit any significant differences. The Chol/phospholipid ratio was increased in obese patient samples (Figure 5F). The latter was due both to a small increase in Chol levels (Figure 5E) and to a decrease in phospholipids, particularly SM- (Figure 5D). The increase in Chol is not statistically significant, but the increase in Chol/phospholipid ratio is. Perhaps this is the result of a compensating effect in the RBC lipids, with the increase in (rigidifying) Chol being countered by a decrease in (also rigidifying) sphingomyelin (SM).

**Figure 6:**
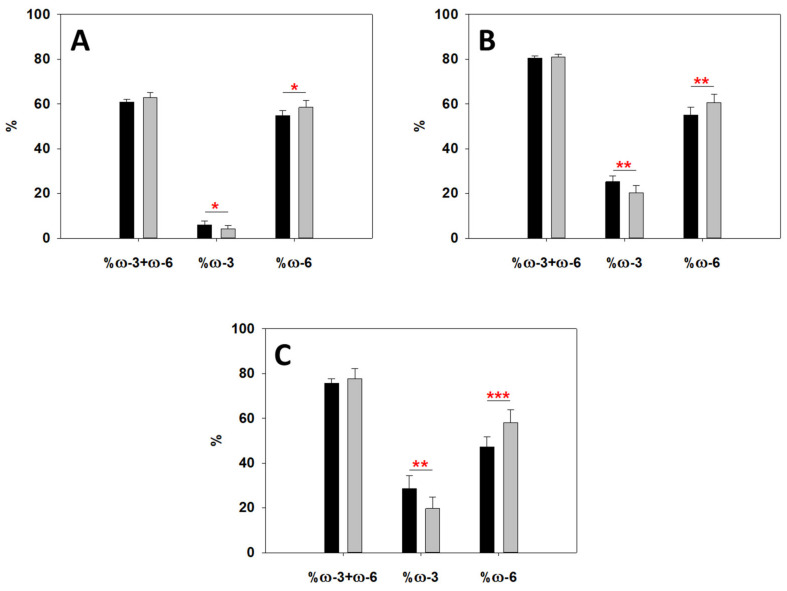
Lipidomic analysis of ω-3 and ω-6 presence in specific lipid species. Percent ω-3 and/or ω-6 in PC (**A**), PE (**B**), and PS (**C**). Black bars refer to control RBC group, while gray bars represent the obese patient RBC group. While total values for combined ω-3 + ω-6 are constant, both a decrease in ω-3 and an increase in ω-6 are detected for each lipid species in obese patient RBC. Average values ± S.D. Significance according to Student’s t-test: (*) *p* < 0.05; (**) *p* < 0.01; (***) *p* < 0.001. *n* = 8.

Lipidomics was also used to evaluate the degree of unsaturation of the different phospholipid species (Supplementary Figure S1) of obese patient RBC and control RBC, but no statistical differences were found between any of them, despite a slight decrease of PS saturation in obese patient RBC. Interestingly, further analysis of the nature of the unsaturated fatty acids for PC, PE, and PS revealed differences in ω-3 and ω-6 lipid profiles (Figure 6). Although the total amount of combined ω-3 + ω-6 remained constant, a significant decrease was detected in ω-3 for obese patient RBC, as well as an equivalent significant increase in ω-6. This confirmed the trend observed above for global fatty acids, and this could be a key explanation for some of the differences detected in membrane stiffness, as will be later assessed in the Discussion section.

### 2.5. Differences in Blood Plasma between Obese Patients and Control Blood Samples

**Figure 7:**
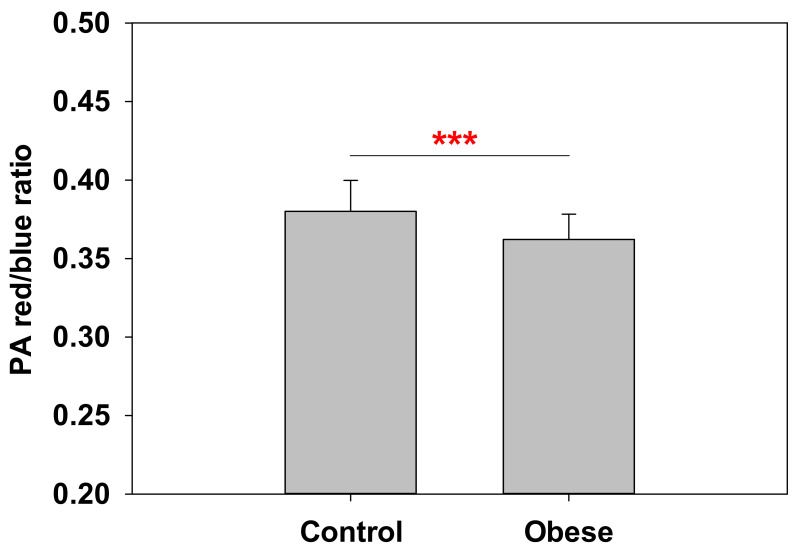
PA probe measurements of blood plasma. Red/blue intensity ratio (RBIR) values for control and obese blood plasma revealed a highly significant reduction in obese patients. Experiments performed at 37 °C (*n* = 35 for control and *n* = 39 for obese). Average values ± S.D. Significance according to Student’s t-test: (***) *p* < 0.001.

Having demonstrated the differences between obese patient RBCs and control RBCs, we performed a final set of experiments exploring the possibilities of additional differences between blood plasma from obese and control patients. Push–pull pyrene (PA) is a fluorescent dye that exhibits different emission spectra depending on the microenvironment, due to an intramolecular energy transfer [29]. It is a member of the so-called solvatochromic probe family. In the case of PA, a fluid environment gives a red-colored emission, while a more ordered area yields a blue-colored one. Thus, the red/blue intensity ratio (RBIR) is generally a marker of fluidity. Although PA has been reportedly used for membranes [29,30], we used this probe to analyze blood plasma and the results are shown in Figure 7. PA red/blue ratios in the range 0.32–0.38 are commonly found in liquid-ordered bilayers, as they exist in erythrocytes [30]. A significant decrease in RBIR was detected for obese patient blood plasma. The interpretation of this result will be extended in the Discussion section, as we would initially not expect a great contribution from lipids in blood plasma due to RBCs and white blood cells being absent, but the probe was sensitive to blood plasma lipoproteins, Chol, and triacylglycerols (TG) (Supplementary Figure S2).

## 3. Discussion

Results from two very different techniques (TMA-DPH anisotropy and AFM) show a clear tendency for obese patient RBC to exhibit a higher fluidity in their membranes, or, more specifically, at the polar–non-polar interface of the membrane bilayers than the control cohort (Figure 2 and Figure 3). This finding may be surprising and even seem counterintuitive at first glance, as obesity is often associated with higher Chol levels as well as saturated lipids in plasma. Both kinds of lipids are known to induce membrane stiffness [25], therefore reducing bilayer fluidity. However, at the membrane level, the opposite appears to be true. Lipidomics did not show any significant increase in Chol nor in saturated lipids. This is relevant because it demonstrates that high levels of Chol or a diet rich in saturated lipids does not necessarily translate exactly to the RBC membrane (in the specific form of cholesterol or saturated lipids, as confirmed by Supplementary Figure S1), but rather it may have a complex effect on RBC metabolism. Still, we should not overlook the slight increase observed in Chol for obese patient RBCs which, despite not being statistically significant, could still have some effect (Figure 5). In fact, Chol/phospholipid ratio was significantly increased in obese patients (Figure 5F), in agreement with the observations by Cazzola et al. [17].

Regarding RBC metabolism of obese patients, global fatty acid lipidomics indicates an increase in arachidonic acid, a ω-6 fatty acid which is a well-described precursor for proinflammatory agents [31] (Figure 4). In accordance, obese patient RBCs also show a significant increase in another rather uncommon ω-6 fatty acid, DGLA (dihomo-γ-linolenic acid) (Figure 4), which is a reported precursor for anti-inflammatory molecules and blocks arachidonic conversion to proinflammatory leukotrienes [32]. The increase in ω-6 fatty acids agrees with previous observations by Cazzola et al. [17]. This points to a putative inflammatory profile for obese patient RBCs, which they try to equilibrate back to homeostasis. This may also be related to the deregulation of the ω-6/ω-3 equilibrium [33]. Pan et al. [15] described an increase in Δ9-desaturase and a concomitant decrease in Δ5-desaturase activities in obese patients that could explain the changes in fatty acid composition mentioned above. This trend in Δ5-desaturase activity was also observed in previous studies in children with overweight and obesity [20]. Regarding the saturated/monounsaturated pathway, obese patients showed a higher SFA/MUFA ratio (Figure 4) due to lower levels of MUFA, as compared to normal weight individuals. As there was not any difference in the intake of any specific fatty acid between normal and obese subjects (according to a nutrition questionnaire, data not shown), the data would suggest a metabolic imbalance in favor of the accumulation of saturated fats and proinflammatory ω-6 fats.

A comparison of both sets of lipidomic data, fatty acids and lipid classes, indicates a generally good agreement. ω-3 fatty acids appear to be less prevalent in obese patient RBCs, as described above, and this is definitely the case also for the specific lipids under study: PC, PE, and PS (Figure 6), in agreement with global ω-3 values for fatty acids, such as DHA (Figure 4). The reduction in ω-3 fatty acids is relevant as they are reportedly linked to cardiovascular health [34], and obesity is a cardiovascular risk factor [35]. The comparison of global vs. specific lipids charts indicates that the differences in ω-6 and ω-3 levels in PC, PE, and PS are more significant than those of global fatty acid levels. This was expected as these three species are prevalent and changes in their fatty acid profiles would definitely have a noticeable effect on membrane biophysics.

An additional factor in obese patient RBC membrane biophysics seems to be the significant reduction in SM, as this lipid class contains mainly saturated fatty acids (this was confirmed by our own experiments in Supplementary Figure S1D) and, in most cases, it acts as a membrane ordering agent. It is also a common partner for Chol as they exhibit a mutual preferential interaction [7], which is the cornerstone of the ‘lipid raft’ hypothesis [36]. Leaving apart the discussion on whether RBC membranes contain any ‘lipid rafts’ (which is a highly debatable issue with conflicting reports [37,38,39,40,41]), the stiffening effect of both SM and Chol for lipid membranes is well known [42,43]. Thus, a reduction of SM levels could also be considered as a possible cause of the increase in membrane fluidity. However, a report from Cazzola et al. [17], using DPH that, unlike TMA-DPH, partitions into the membrane hydrophobic core, observed that the hydrophobic region of membranes was more rigid, or less fluid, in apparent contradiction with our results. (It should also be noted that the average BMI of ‘obese’ patients in Cazzola et al. [17] was 29.3, in contrast with 38–41 in the present study.)

The change in membrane fluidity cannot be fully ascribed to the decrease of SM levels: lower SM levels would cause a smaller amount of saturated fatty acids (those attached to SM), and this would in turn cause a decrease in membrane order as observed by DPH, due to a reduction in bilayer packing, but DPH-based reports point to an opposite effect, as previously mentioned [17]. Therefore, there must be an additional reason that explains the higher obese patient RBC membrane interfacial fluidity. Here, we propose that the switch from ω-3 to ω-6 lipids (particularly in those prevalent lipid classes such as PC, PE, and PS) is the main cause for the effect. This is initially supported by published reports indicating that ω-6 fatty acids generate a higher membrane fluidity than ω-3, due to an increase in area per lipid [44]. This is caused by the double bond being closer to the carboxyl in the fatty acid, which causes a longer bend (also called ‘kink’) of the *cis*-bond, occupying more space than the ω-3, which in turn exhibits a shorter ‘kink’. If we consider our results, this explanation fits well with TMA-DPH (Figure 2) data, as TMA-DPH places itself close to the polar headgroups.

Regarding AFM results, the molecular mechanisms involved in an indentation process of a lipid membrane, despite being a widespread tool for nanomechanical characterization [43,45,46,47], are not as obvious as they may seem at first glance. Two main hypotheses were proposed [48]: (i) an elastic compression of the bilayer until the force achieves a critical point where it is broken as a whole (i.e., in a single step), and (ii) a sequential model where, after an initial bilayer compression, the tip encounters different parts of the bilayer, which act as distinct ‘layers’ or barriers, and the tip advances through them step by step. The classic approach leaned towards the first hypothesis because most of the AFM bilayer breakthrough events were apparently in a single step, with some exceptions caused by bilayer uncoupling of the leaflets [24,49,50]. However, recent reports and the use of advanced force spectroscopy techniques (such as force-clamp spectroscopy) point to a sequential model even in the simplest ‘coupled’ models [51]. Therefore, changes in the breakthrough forces of different membrane samples may be caused not only by lipid packing or molecular order of the hydrophobic region (as commonly thought), but also by changes to other ‘layers’ across the membrane, including the interphase between polar headgroups and lipid tails. In this case, a destabilization of the interphase by an increased presence of ω-6 lipids would cause a lower breakthrough force and a reduction in nanomechanical resistance, even if reports indicate that the hydrophobic region measured by DPH would be more ordered [17]. An additional explanation for this is that the tip encounters the interphase before the hydrophobic region, and a destabilized interphase may facilitate tip advancement during the indentation process, having a greater impact on the force required for the whole process. Thus, AFM results are compatible with the hypothesis of an interphase-driven effect.

Note that alterations in RBC membrane proteins (level, isoforms, interaction with cytoskeleton) might contribute to the observed changes in nanomechanical properties. This is why, in addition to the physical measurements, we performed lipidomic analyses. Changes in membrane proteins might contribute to nanomechanical effects, but we can confirm that most of the physical changes can be explained in terms of modifications in lipid composition.

Results regarding the use of PA probes for blood plasma might be interesting. While the use of PA is well documented for membranes and the RBIR values are indicative of bilayer order [29,30], blood plasma does not exhibit a significant lipid organization as there are no cellular elements present, with the exception of platelets. Considering that lipid quantities from platelets are not sufficient for an adequate PA signal, additional experiments were required for a better understanding of PA behavior. For this purpose, the relationship between PA and blood plasma lipoproteins carrying cholesterol (namely, HDL and LDL) was explored (Supplementary Figure S2). Experiments showed that RBIR values for PA decreased when HDL/LDL ratio was lower, indicating a reduction in HDL and/or an increase in LDL. Lower HDL/LDL ratios are a well-known marker of cardiovascular disease risk [52,53]. Thus, PA indicates that obese patients have significantly lower HDL/LDL ratios and, presumably, a higher cardiovascular disease risk. This kind of assay could also be of clinical interest to improve HDL/LDL ratio measurements. In addition, PA probe was also sensitive to total Chol+TG levels, which in turn may also have further clinical interest as high levels of chylomicrons (TG carrier lipoprotein) are related to cardiovascular risk and/or acute pancreatitis [54].

As a note of caution, it should be taken into consideration that the sample population was not evenly distributed by gender or age, as the female population was more prevalent in both obese and control groups, and obese patients were generally older. This is relevant because it limits our capacity to make further analysis of our results but opens the possibility for future experiments to evaluate the impact of gender or age in obese patient RBC properties. In fact, a previous publication from this consortium showed some differences in the fatty acid profile of RBC membranes when children with obesity was compared to adults with obesity, highlighting that children with obesity presented higher levels of ω-6 polyunsaturated FAs (mainly linoleic acid) and lower values of ω-3 FAs (mainly DHA) compared with adults with obesity [55].

In conclusion, our data offer an integrated scenario of molecular factors connected with obesity, obtained by a combination of RBC membrane lipidome analyses (fatty acids and lipid classes) with the RBC membrane and blood plasma biophysical properties measured by different tools. We have observed that, in obese adults, the polar–non-polar interface in red blood cell membranes is more fluid (or less ordered) and more easily penetrable than in non-obese subjects. The use of two techniques based on very different physical properties, such as fluorescence and nanomechanics, lends credibility to our observation, which is also in agreement with the observed changes in fatty acid composition between the two groups of samples. The ω-6/ω-3 balance increase is arguably the basis of the changes of membrane properties here observed by biophysical measurements.

## 4. Materials and Methods

### 4.1. Subjects and Study Design and Anthropometric Measurements

Bodyweight (kg) and height (cm) were measured by standard methods. Body mass index (BMI) was calculated as weight (kg) divided by the square of the height (m^2^). Anthropometric parameters, as well as blood sampling, were performed by specialists during the participant’s visit to the Hospital Universitario Cruces/IIS Biocruces Bizkaia after acceptance to participate in the study and signing of informed consent.

### 4.2. Blood Management and RBC Isolation

Blood samples from informed subjects were obtained by venipuncture at the Endocrinology Service of the Cruces University Hospital (Barakaldo, Spain). Blood was collected in BD Vacutainer tubes with 18.0 mg of EDTA (BD Vacutainer Systems, Franklin Lakes, NJ, USA) and was transported refrigerated to the Biofisika Institute (or to Fundación AZTI for global fatty acid lipidomics) on the same day of extraction. Red blood cells were isolated and washed using sequential centrifugations [56]. Finally, the washed RBCs were diluted to 10^8^ cell/mL in assay buffer: 25 mM Hepes, 150 mM NaCl, 1 mM EDTA, pH 7.4.

### 4.3. Fluorescence Anisotropy of TMA-DPH in Erythrocyte Membrane

For RBC staining, 5 μM TMA-DPH (in DMSO) was added to 10^8^ cells/mL washed red blood cells. Final DMSO concentration was 7.8 µg/mL. The system was left incubating 90 min at room temperature.

Fluorescence anisotropy measurements were performed using a FluoroMax-3 spectrofluorometer (Horiba Jobin Yvon, Edison, NJ, USA) with polarizers in the excitation and emission channels and equipped with type L measurement system. The instrument software computes anisotropies for each experimental point, automatically correcting for the G factor. The FluoroMax-3 was equipped with thermoregulated holders that allowed measures at 20 °C and 37 °C. The wavelengths of excitation and emission for TMA-DPH were 360 and 430 nm, respectively. To avoid light scattering and inner filter effects, fluorescence anisotropy was measured on increasingly diluted samples. Only when anisotropy values remained constant with further dilution were they recorded.

### 4.4. Sample Preparation for AFM Measurements

RBCs were prepared for AFM analysis with an optimized version of a protocol from a previous work [37]. First, RBC were isolated from the rest of blood samples by centrifugation as described above, but using a particular ‘RBC Buffer’, with the following composition: 32 mM HEPES, 125 mM NaCl, 1 mM MgSO_4_, 1 mM CaCl_2_, 5 mM KCl, 5 mM glucose, at pH 7.2. This buffer helped with RBC viability during sample preparation and AFM measurements [37]. For AFM experiments, RBC concentration was diluted 100-fold to a concentration of ~10^6^ cell/mL, as the technique requires a reduced amount of cells, with clear separation from each other.

Round glass coverslips were washed with ethanol (analytical grade) and mounted onto a CoverslipHolder (JPK Instruments, Berlin, Germany). Ethanol washing is reportedly a non-invasive method to achieve enough RBC adhesion in the absence of fixatives [37]. After 30 min to ensure complete ethanol evaporation, the RBC diluted solution was added to the coverslip and left for 45 min to optimize cell adhesion.

### 4.5. AFM Force Measurements

RBC nanomechanical resistance to indentation was measured by force spectroscopy (AFM), in order to evaluate the force necessary to completely pierce the cell. RBCs were analyzed with a JPK NanoWizard 2 AFM (JPK Instruments, Berlin, Germany) mounted onto a Leica DMI4000B epifluorescence microscope (Leica Microsystems, Wetzlar, Germany) on a Halcyonics Micro 40 anti-vibration table (Halcyonics, Inc., Menlo Park, CA, USA) and inside an acoustic enclosure (JPK Instruments). This setup allowed direct observation of RBC with light mode through a 40X objective, so that the AFM tips could be conveniently placed on top of each RBC. Force curves were performed with V-shaped MLCT silicon nitride cantilevers (Bruker AXS, Karlsruhe, Germany), with spring constants of 0.01 and 0.03 N/m, calibrated just before the experiments with the thermal noise method. No images were taken of RBC as this would require the use of fixatives, therefore altering the nanomechanical profile of cells.

Force curves were performed in the conventional force-extension mode with the following specifications: tip speed = 1 μm/s, Z-length = 2 μm, piezo range = 12 μm, to a target force (setpoint) of 10 nN. The curves were directed at the center of the RBC (the thinnest part of the cell). The number of curves for each sample was 50–75 curves, with typically no more than 4 curves performed in the same cell, to avoid any effect caused by any possible damage or inefficient cell recovery. Force curves were later batch-analyzed with the JPK Data Processing software in order to quantify the breakthrough events, then a mean value and a standard deviation was obtained for every sample. It is important to consider that the force curve in the vast majority of cases was able to pierce through the complete cell, therefore two consecutive breakthrough events were typically detected for each force curve. AFM measurements were done at room temperature.

### 4.6. PA Ratio in Blood Plasma Using Fluorescence Spectroscopy

Blood plasma was obtained by centrifuging the blood for 4 min at 3000× *g* and 4 °C, the pellet was discarded and the supernatant was collected. A 1/16 dilution of the blood plasma was made with assay buffer and 0.005 μM PA (in DMSO) was added. One hour of incubation was allowed before measuring in the spectrofluorometer.

The fluorescence spectra of the PA probe were measured on a QuantaMaster 40 spectrofluorometer (Photon Technology International, Lawrenceville, NJ, USA), with a previously described method [30]. Emission spectra were collected between 450 and 700 nm, exciting at 430 nm for PA, using a 430 nm bandpass filter to minimize detection of dispersed light. A thermal TC125 controller (Quantum Northwest, Liberty Lake, WC, USA) was used to stabilize the sample temperature at 37 °C. Once the emission spectra were obtained, in order to calculate the Red/Blue Intensity Ratio (RBIR), the areas of the blue (500–530 nm) and red (573–613 nm) regions were measured with the software PTI Felix-GT software (Photon Technology International, Lawrenceville, NJ, USA).

### 4.7. Mature RBC Lipidomics of Global Fatty Acids

The fatty acid composition of mature RBC membrane phospholipids was obtained from blood samples (approximately 2 mL) collected in vacutainer tubes containing ethylenediaminetetraacetic acid (EDTA). Samples were shipped to the Lipidomic Laboratory (Bologna, Italy) at ambient temperature and, upon arrival, underwent quality control for the absence of hemolysis. During the blood work-up, before lipid extraction and lipid transesterification to fatty acid methyl esters (FAMEs), the automated protocol includes the selection of mature RBCs, as reported previously [20,57,58]. Briefly, whole blood in EDTA was centrifuged (4000 revolutions per minute (rpm) for 5 min at 4 °C) and the mature cell fraction was isolated based on the higher density of the aged cells [59] and controlled by the use of a cell counter (Scepter 2.0 with Scepter™ Software Pro, EMD Millipore, Darmstadt, Germany). All the subsequent steps were automated and included cell lysis, isolation of the membrane pellets phospholipid extraction from pellets using the Bligh and Dyer method [60], transesterification to FAMEs by treatment with a potassium hydroxide (KOH)/methyl alcohol (MeOH) solution (0.5 mol/L) for 10 min at room temperature, and extraction using hexane (2 mL). The FAMEs were analyzed using capillary column gas chromatography (GC). GC analysis was run on the Agilent 6850 Network GC System (Agilent, USA), equipped with a fused silica capillary column Agilent DB23 (60 m × 0.25 mm × 0.25 μm) and a flame ionization detector. Optimal separation of all fatty acids and their geometrical and positional isomers was achieved. Identification of each fatty acid was made by comparison to commercially available standards and to a library of *trans* isomers of MUFAs and PUFAs. The amount of each FA was calculated as a percentage of the total FA content (relative %), with more than 97% of the GC peaks recognized with appropriate standards.

### 4.8. Mature Red Blood Cell Membrane Fatty Acid Cluster

Twelve FAs were chosen as a representative cluster of the main building blocks of the RBC membrane glycerophospholipids and of the three FA families (SFA, MUFA, and PUFA): for SFAs, palmitic acid (C16:0) and stearic acid (C18:0); for MUFAs, palmitoleic acid (C16:1; c9), oleic acid (C18:1; c9), and *cis*-vaccenic acid (C18:1; c11); for ω-3 PUFAs, eicosapentaenoic acid (EPA) (C20:5) and docosahexaenoic acid (DHA) (C22:6); for ω-6 PUFAs, linoleic acid (LA) (C18:2), dihomo-gamma-linolenic acid (DGLA) (C20:3), and arachidonic acid (AA) (C20:4); for geometrical *trans* fatty acids (TFAs): elaidic acid (C18:1 t9) and mono-*trans* arachidonic acid isomers (mono-*trans*-C20:4; ω-6 recognized by standard references as previously described by Ferreri et al. [61]). Considering these fatty acids, different indexes previously reported in the literature [20] were calculated: Omega-3 ω-3 Index: (%EPA + %DHA) an index suggested as a cardiovascular disease risk factor; (%SFA/%MUFA) index related with membrane rigidity; inflammatory risk index (% ω-6)/(% ω-3); PUFA balance (%EPA + %DHA)/total PUFA × 100; free radical stress index (sum of *trans*-18:1 + summary (Σ) of mono-*trans* 20:4 isomers); unsaturation index (UI) (%MUFA) + (%LA/2) + (%DGLA/3) + (%AA/4) + (%EPA/5) + (%DHA/6); peroxidation index (PI) (%MUFA/0.025) + (%LA) + (%DGLA/2) + (%AA/4) + (%EPA/6) + (%DHA/8). Additionally, the enzymatic indexes of elongase and desaturase enzymes, the two classes of enzymes of the MUFA and PUFA biosynthetic pathways, were inferred by calculating the product/precursor ratio of the involved FAs.

### 4.9. RBC Lipidomics of Specific Lipid Species

#### 4.9.1. Sample Preparation

For lipidomic experiments, 8 samples were randomly selected from each group, 16 samples in total. The washed RBCs were centrifuged at 150,000× *g* (4 °C, 1 h) in an Ultra Optima L100K ultracentrifuge (Beckman Coulter, 357656, Brea, CA, USA). The supernatant was discarded, and the pellet was collected. The pellet was resuspended in 750 μL of assay buffer. Four volumes (3000 μL) of cold isopropanol (at −20 °C) were added, in 3 samples the internal standard mixtures Splash Lipidomix and Cer/SPH Mixture I from Avanti Polar Lipids were added. The mixture was vortexed for 1 min and incubated 10 min at room temperature to enhance protein precipitation. Then, it was centrifuged at 13,000× *g* (20 min, 4 °C). The supernatant was transferred to a glass vial and its phospholipid concentration was calculated by the fiske method [62]. Once the concentration had been calculated, 100 nmoles of phospholipid from each sample were brought to dryness in a speed-vac at room temperature and stored at −80 °C until their analysis.

#### 4.9.2. Analysis of the Samples

Global lipidomic profiles were determined by tandem MS using an electrospray ionization source (ESI) in negative (−) and positive mode (+) after separation of lipid classes by a reverse-phase ultrahigh performance liquid chromatography (UHPLC). The chromatographic separation was achieved on a Vanquish UHPLC system (ThermoFisher Scientific, Waltham, MA, US), equipped with a binary solvent delivery pump, a thermostated autosampler, and a column oven. A reverse-phase column (Acquity UPLC C18 CSHTM 2.1 mm × 100 mm, 1.7 µm) and a pre-column (Acquity UPLC C18 CSHTM 2.1 mm × 5 mm, 1.7 µm: VanGuard) were used at 65 °C to separate individual lipids. The mobile phases consisted of acetonitrile and water (40:60, *v/v*) with 10 mM ammonium formate and 0.1% formic acid (phase A), and acetonitrile and isopropanol (10:90, *v/v*) with 10 mM ammonium formate and 0.1% formic acid (phase B). The applied elution conditions were 0–2 min, 40–43%B; 2–2.1 min, 43–50 %B; 2.1–12, 50–54%B, 12–12.1 min, 54–70 %B; 12.1–18 min, 70–100 %B; finally, washing and reconditioning of the column was done. The flow rate was 500 μL/min, and the injection volume was 10 μL. All samples were kept at 10 °C during the analysis.

All UHPLC-MS/MS data were acquired on a Q Exactive HF-X hybrid quadrupole-Orbitrap mass spectrometer (ThermoFisher Scientific, USA) equipped with a HESI (heated electrospray ionization) source using a data-dependent LC-MS/MS method (top 15 MS2) in both positive mode and negative mode. The mass spectrometer settings were optimized using the SplashTM LipidoMixTM and Ceramide/Sphingoid standard internal mixture (Table 2). The flow rates of sheath gas, sweep gas, and auxiliary gas for both polarities were adjusted to 35, 0, and 10 (arbitrary units). For both ionization modes, the capillary temperature and the heater temperature were maintained at 285 °C and 370 °C, respectively, while the spray voltage was 3.90 KV for positive and 3.20 kV for negative ionization. The S-lens RF level was set at 40. The Orbitrap mass spectrometer was operated at a resolving power of 120,000 in full-scan mode (scan range: 250–2000 *m*/*z*, automatic gain control target 10^6^ and 7500 in Top15 data-dependent MS2 mode (HCD fragmentation with a stepped normalized collision energy of 25 and 30 in positive mode, and 20, 30, and 40 in negative ion mode; injection time 11 ms; isolation window 1 *m*/*z*; automatic gain control target 10^5^ with a dynamic exclusion setting of 6.0 s). The spectrometer was calibrated externally every three days within a mass accuracy of 1 ppm.

#### >4.9.3. Data Processing

All the MS data were acquired and processed using the Xcalibur 4.1 software package, while the LipidSearch software version 4.2.27 (Mitsui Knowledge Industry, Tokyo, Japan) was used to identify and quantify the lipid species in these complex biological samples. The key processing parameters were target database, General; precursor tolerance, 5 ppm; product tolerance, 5 ppm; product ion threshold, 1%; m-score threshold, 2; Quan *m*/*z* tolerance, ±5 ppm; Quan RT (retention time) range, ±0.5 min; use of main isomer filters and ID quality filters A, B, C, and D; Adduct ions H^+^, Na^+^ and NH4^+^ for positive ion mode, and H^−^ and HCOO^−^ for negative ion mode. The lipid classes selected for the search were: LPC (lysophosphatidylcholine), PC (phosphatidylcholine), LPE (lysophosphatidylethanolamine), PE (phosphatidylethanolamine), LPG (lysophosphatidylglycerol), PG (phosphatidylglycerol), LPI (lysophosphatidylinositol), PI (phosphatidylinositol), LPS (lysophosphatidylserine), PS (phosphatidylserine), LPA (lysophosphatidic acid), PA (phosphatidic acid), SM (sphingomyelin), Cer (ceramide), Hex1Cer (hexosylceramide), Hex2Cer (dihexosylceramide), Hex3Cer (trihexosylceramide), SPH (shingosine), CL (cardiolipin), MG (monoacylglycerol), DG (diacylglycerol), TG (triacylglycerol), ChE (cholesterol ester), FA (fatty acid), and AcCa (acylcarnitine)

Quantification was carried out by normalization of the extracted monoisotopic ion peak area of each native lipid species to the intensity of the extracted monoisotopic ion peak area of the internal standard. The internal standards used in this study were chosen to avoid those present in plasma samples.

## 5. Conclusions

Our study supports the hypothesis that obesity influences the composition and properties of plasma membranes in cells other than adipocytes. Specifically, the data demonstrate a significant decrease in the fluidity (increased order) of the polar–non-polar interfacial region of RBC from obese patients when compared with a control group. This affects membrane order measured by TMA-DPH anisotropy and stiffness measured by AFM force spectroscopy. Lipidomics of the samples points to a deregulation of lipid homeostasis for obese RBC, with significant changes in concentration of ω-3 and ω-6 fatty acids. More precisely, obese patient RBCs undergo an increase is some ω-6 fatty acids such as arachidonic acid, while reducing ω-3 ones, such as DHA. This ‘switch’ from ω-3 to ω-6 fatty acids in obese patient RBC membranes is also detected for abundant specific lipid species such as PC, PE, and PS. In addition, a significant reduction in SM is detected for obese patient RBC. Both events, SM reduction and, perhaps more decisively, the increase of ω-6 fatty acids seem to contribute to the aforementioned fluidity of obese patient RBC membranes. Finally, experiments on blood plasma with PA probe pointed to a reduction in the HDL/LDL ratio for obese patients, which is a relevant marker for cardiovascular disease risk.

## Figures and Tables

**Table 1:** Physical characteristics of obese and control subjects (mean ± SD).

	Sex	Age (yr)	BMI (kg × m^−2^)
Control (49)	16 M	30.2 ± 13.2	21.8 ± 5.6
	33 F	34.5 ± 14.5	21.5 ± 4.2
Obese (52)	16 M	48.0 ± 13.0	38.2 ± 11.0
	36 F	45.8 ± 12.6	40.7 ± 8.7

**Table 2:** Lipid composition of the internal standard mix.

Internal Standard Mix	Compound Name	Exact Mass	Chemical Formule	Conc. (µg/mL)
Splash LipidoMix	15:0–18:1(d7) PC	752.6061	C_41_H_73_D_7_NO_8_P	150.6
	15:0–18:1(d7) PE	710.5591	C_38_H_67_D_7_NO_8_P	5.3
	15:0–18:1(d7) PS (Na Salt)	776.5309	C_39_H_66_D_7_NNaO_10_P	3.9
	15:0–18:1(d7) PG (Na Salt)	763.5357	C_39_H_67_D_7_NaO_10_P	26.7
	15:0–18:1(d7) PI (NH_4_ Salt)	846.5963	C_42_H_75_D_7_NO_13_P	8.5
	15:0–18:1(d7) PA (Na Salt)	689.4994	C_36_H_61_D_7_NaO_8_P	6.9
	18:1(d7) Lyso PC	528.3921	C_26_H_45_D_7_NO_7_P	23.8
	18:1(d7) Lyso PE	486.3451	C_23_H_39_D_7_NO_7_P	4.9
	18:1(d7) Chol Ester	657.6441	C_45_H_71_D_7_O_2_	329.1
	18:1(d7) MG	363.3366	C_21_H_33_D_7_O_4_	1.8
	15:0–18:1(d7) DG	587.5506	C_36_H_61_D_7_O_5_	8.8
	15:0–18:1(d7)-15:0 TG	811.7646	C_51_H_89_D_7_O_6_	52.8
	D18:1-18:1(d9) SM	737.6397	C_41_H_72_D_9_N_2_O_6_P	29.6
	Cholesterol (d7)	393.3988	C_27_H_39_D_7_O	98.4
Ceramide/Sphingoid Internal Standard Mixture I	Sphingosine (C17)	285.2668	C_17_H_35_NO_2_	6.9
	Sphinganine (C17)	287.2824	C_17_H_37_NO_2_	7.1
	Sphingosine-1-P (C17)	365.2331	C_17_H_36_NO_5_P	9.3
	Sphinganine-1-P (C17)	367.2488	C_17_H_38_NO_5_P	9.2
	Ceramide (C12)	481.4495	C_30_H_59_NO_3_	11.6
	Ceramide (C25)	663.6529	C_43_H_85_NO_3_	16.2
	Ceramide-1-P (C12)	561.4158	C_30_H_60_NO_6_P	13.4
	Sphingomyelin (C12)	646.5050	C_35_H_71_N_2_O_6_P	15.7
	Glucosyl(β) C12 Ceramide	643.5023	C_36_H_69_NO_8_	15.6
	Lactosyl(β) C12 Ceramide	805.5550	C_42_H_79_NO_13_	19.9

## Data Availability

The data presented in this study are available on request from the corresponding author.

## Supplementary Materials

The following are available online at https://www.mdpi.com/article/10.3390/ijms23031920/s1.
