# Supplementary material for: Erythrocyte Membrane Nanomechanical Rigidity Is Decreased in Obese Patients
Source: Int J Mol Sci. 2022 Feb 8;23(3):1920. doi: 10.3390/ijms23031920 (PMC8836476; doi:10.3390/ijms23031920)
Supplement: Supplementary file 1 [file ijms-23-01920-s001.zip › ijms-1586360-Supplementary.pdf]

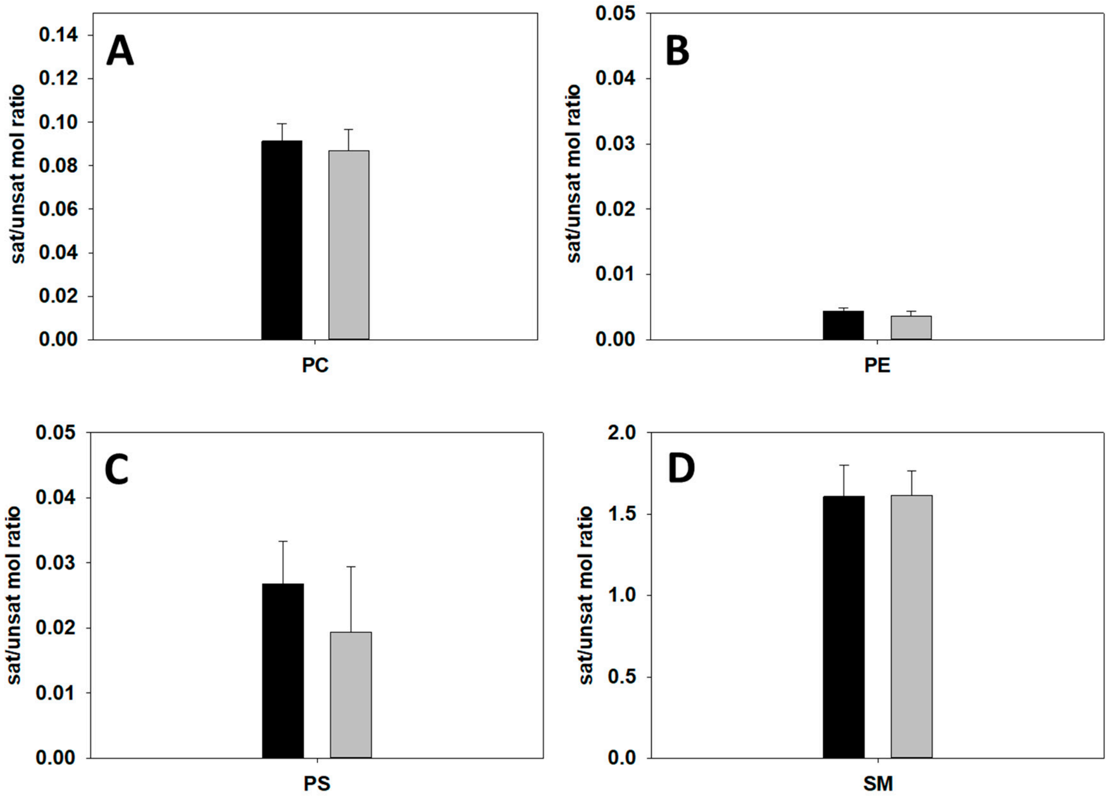

Figure S1. Saturated/unsaturated mol ratio for specific lipid species. Species studied were PC (A), PE (B), PS (C) and SM (D). Black bars refer to control RBC group, while grey bars represent the obese patient RBC group. No significant differences were observed.

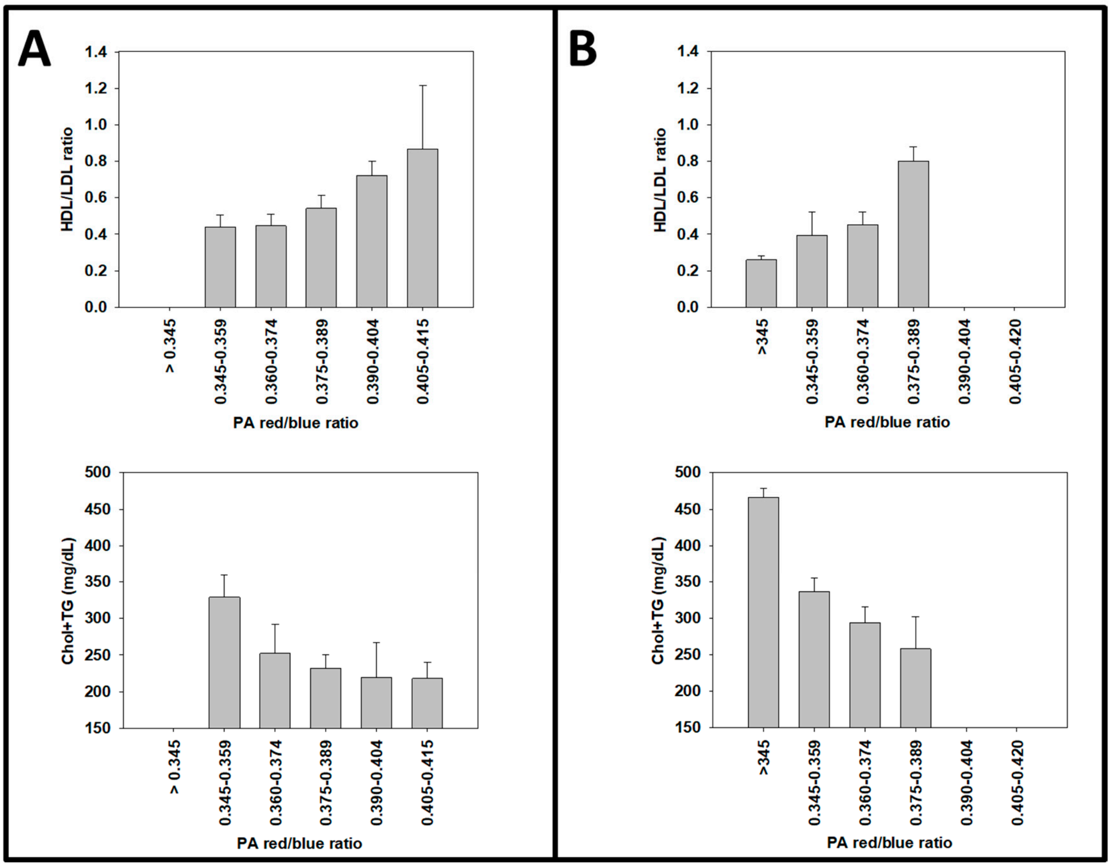

Figure S2. PA probe fluorescence measurements of blood plasma samples. The bar plots represent different intervals of PA RBIR ratio for blood plasma samples (X-axis), in relation to the HDL/LDL ratios (top) and cholesterol + triglycerides (Chol+TG) (bottom) values (Y-axis), the latter obtained from the clinical samples. Left-hand panels (A), control group. Right-hand panels (B), obese patients (n = 16 for each sample group). RBIR values are directly related to HDL/LDL ratios, and inversely related to Chol+TG values.
